# Novel Study on N-Nitrosamines as Risk Factors of Cardiovascular Diseases

**DOI:** 10.1155/2014/817019

**Published:** 2014-08-27

**Authors:** Salah A. Sheweita, Heba A. El-Bendery, Mostafa H. Mostafa

**Affiliations:** ^1^Department of Biotechnology, Institute of Graduate Studies & Research, Alexandria University, 163 Horreya Avenue, P.O. Box 832, EL-Chatby, Alexandria 21526, Egypt; ^2^Department of Environmental Studies, Institute of Graduate Studies & Research, Alexandria University, 163 Horreya Avenue, P.O. Box 832, EL-Chatby, Alexandria 21526, Egypt

## Abstract

Millions of people are exposed daily to N-nitrosamines from different environmental sources. The present study aims at investigating the role of N-nitrosamines in the alteration of homocysteine, lipid profile, oxidative stress, paraoxonase activity, antioxidant enzymes, and free radicals which are important risk factors for CVD. In addition, biomarkers of cardiovascular diseases such as creatine kinase MB activity (CK-MB) and lactate dehydrogenase (LDH) as well as protein expression of both glutathione peroxidase and glutathione S-transferase *π* isozyme were assayed after treatment of rats with 0.2 mg/kg body weight of N-nitrosodibutylamine (NDBA), N-nitrosoethylbutylamine (NEBA), N-nitrosobutylpropylamine (NBPA), N-nitrosodiethylamine (NDEA), N-nitrosodimethylamine (NDMA), and N-nitrosodiphenylamine (NDPA) as a daily dose for two weeks. LDL levels, paraoxonase activity, reduced glutathione levels, and glutathione reductase activities were increased, whereas HDL levels decreased after treatment of rats with most of N-nitrosamines compared to control group. Moreover, levels of free radicals and catalase activity increased, whereas protein expression of both glutathione peroxidase and glutathione S-transferase decreased after treatment of rats with some N-nitrosamines. The data showed that most N-nitrosamines increased CK-MB and LDH activities. It is concluded that N-nitrosamines increased levels of free radicals, and decreased the activity of antioxidant enzymes which may consequently increase the incidence of CVDs.

## 1. Introduction

N-Nitrosamines are mutagenic and carcinogenic chemicals and are present in large quantities in tobacco smokes [1], pacifiers and baby bottle nipples [2], and cured meats and smoked fish [3]. In addition, N-nitrosamines can be formed endogenously from interaction of nitrate, nitrite with secondary or tertiary amines and amides in human stomach [4]. Moreover, a variety of over-the-counter drugs, food additives, cosmetics, and many agricultural chemicals have been identified as having secondary or tertiary amine or amide groups in their structure that can react with nitrite to form nitrosamines and nitrosamides from simulated human gastric conditions [5–10]. Furthermore, nitrosation of drugs with tertiary amines or amides resulted in the production of known carcinogens [11]. Recently, it has been found that prenatal exposure to nitrosatable drugs may be associated with several congenital malformations, especially with higher nitrite intake [12]. Various N-nitrosamines have been observed to cause abnormal development through DNA alkylation of target organs [13]. A frog embryo exposed to N-nitrosamines was found to develop severe heart defects [14]. In rats, maternal exposure to such compounds resulted in increased incidence of limb malformations, neural tube defects, microcephalus, and hydrocephalus [15, 16].

Prevalence of coronary artery atherosclerosis is increasing worldwide on an annual basis and is the leading cause of millions of deaths in the Western world. The number of people who die from CVDs will increase to reach 23.3 million by 2030 [17, 18]. Hypercholesterolemia, hypertension, smoking, diabetes, and oxidative stress have been identified as risk factors for atherosclerosis development [18]. In addition, many genetic factors are involved in the development of CVDs [19]. Although the outstanding pathological feature of atherosclerosis is the collection of cholesterol esters from circulating lipoproteins in the intimal layer of large arteries, it remains relatively poor predictor of CVDs. Oxidized LDL [LDLoxid] has been reported to be a trigger of atherogenic events which induce proinflammatory molecules that lead to an increase in recruitment of inflammatory cells to the artery wall [20]. HDL, on the other hand, is antiatherogenic, and its protective effects have been ascribed primarily to its ability to shuttle excess cholesterol from peripheral tissues [21, 22]. These protective effects of HDL have been attributed to the presence of various proteins associated with HDL in the circulation including apolipoprotein AI, lecithin cholesteryl-acyltransferase [LCAT], and serum paraoxonases (PONs) [19]. Paraoxonase is synthesized in the liver and in serum is almost exclusively associated with HDL. The enzyme paraoxonase-1 (PON-1) contributes to the antiatherogenic effects of HDL. The antiatherogenic potential of paraoxonase is derived from its capacity to hydrolyze oxidized lipids, phospholipids, cholesterol ester, and hydroperoxides, thus preventing them from accumulating in LDL particles [23].

Several pathophysiological mechanisms involved in the development of atherosclerosis are well known [19]. One of these mechanisms, it has been found that reactive oxygen species or free radical-induced oxidation of lipoproteins may be an important event in this process [19]. Human exposure to different sources of reactive oxygen species [ROS] leads to oxidative stress. Oxidative stress can be generated from secretions by phagocytic white blood cells, dysfunctional endothelial cells or from the autooxidation of catecholamines as the extracellular sources of ROS, while impaired mitochondrial reduction of molecular oxygen is the intracellular source of ROS [24]. In addition, ROS may also result from cellular injury due to exposure to ionizing radiation, ultraviolet rays, cigarette smoking, or other air pollutants and toxic chemicals [25, 26]. Oxidative stress plays an important role in the progression of a number of diseases including atherosclerosis, ischemia-reperfusion injury, hypertension, catecholamine-induced cardiomyopathy, hypertrophy, and congestive heart failure [27]. Excessive production and/or inadequate removal of reactive oxygen species, especially superoxide anion (O_2_^•−^), have been implicated in the pathogenesis of many cardiovascular diseases by decreasing nitric oxide (NO) bioactivity [28]. Antioxidant enzymes as superoxide dismutase (SOD), catalase, glutathione peroxidase, glutathione reductase, and glutathione S-transferase and nonenzymatic antioxidants as glutathione, vitamin C, and vitamin E have been involved in scavenging of free radicals from different tissues [29, 30].

No previous studies have been conducted on the role of N-nitrosamines as risk factors for the incidence of cardiovascular disease since these compounds are present in many different environmental sources. Therefore, the present study was planned to investigate their effects on the different biochemical parameters as lipid profiles, paraoxonase, homocysteine levels, glutathione levels, free radicals, glutathione S-transferase, glutathione peroxidase, and catalase activities as well as other cardiovascular biomarkers such as creatine kinase MB and lactate dehydrogenase in plasma and livers of male rats.

## 2. Materials and Methods

### 2.1. Chemicals

Diethylnitrosamine, dibutylnitrosamine, ethylbutylnitrosamine, propylbutylnitrosamine, diphenylnitrosamine, dimethylnitrosamine, sodium dithionite, nicotinamide adenine dinucleotide phosphate reduced (NADPH) sodium salt, sulfosalicylic acid, bis-(3-carboxy-4-nitrophenyl)-disulfide, 1-chloro-2,4-dinitrobenzene, and all other chemicals were purchased from Sigma Chemical Co., St. Louis, MO, USA.

### 2.2. Animals

Seventy male Sprague-Dawley rats weighing 200–220 g were obtained from the animal house of the Faculty of Medicine, Alexandria University, Alexandria, Egypt. Rats were housed in standard cages and given food and water. The animals were divided into seven groups. The first group (10 rats) was kept as control and received distilled water. The other six groups (10 rats each) were given 0.2 mg/kg as a daily dose of N-nitrosodibutylamine, N-nitrosoethylbutylamine, N-nitrosobutylpropylamine, N-nitrosodiethylamine, N-nitrosodimethylamine, and N-nitrosodiphenylamine for two weeks. At the end of the experimental period, rats were sacrificed by cervical decapitation after administration of diethyl ether as anesthetic, and fasting blood samples were collected in heparinized tubes. Plasma samples were obtained by centrifugation at 4000 rpm for 20 min and stored at –80°C until use.

### 2.3. Enzyme Assessments

At the designated time point, rats were anesthetized with ether and the thoracic cavity was opened for whole body. Liver tissues were vigorously washed in an iced solution of 0.25 M sucrose, which contained 0.001 M EDTA, to avoid contamination from erythrocyte-containing enzymes. Liver tissues were homogenized in 3 volumes of 0.1 M phosphate buffer (pH 7.4) and centrifuged at 12000 ×g for 20 min at 4°C. Reduced glutathione level was estimated in homogenate supernatant of liver tissue using sulfosalicylic acid for protein precipitation and bis-(3-carboxy-4-nitrophenyl)-disulfide for color development, Mitchell et al., 1973 [31]. Glutathione reductase activity was assayed in the supernatant of liver tissue homogenate by monitoring the oxidation of NADPH at 340 nm using the method of Suojanen et al., 1980 [32]. A unit of enzyme activity represents 1 nmol of NADPH oxidized/min per mg protein. GST activity was determined according to the method of Lee et al., 1981 [33]. The conjugate of GSH with 1-chloro-2,4-dinitrobenzene (CDNB) was measured at 340 nm using a double beam spectrophotometer. A unit of enzyme activity is defined as the amount of enzyme that catalyzes the formation of 1 mmol of CDNB conjugate/mg protein/min under the assay conditions. Calculations were made using a molar extinction coefficient of 9.6 mM^−1 ^cm^−1^. The protein concentration was measured by the method of Lowry et al. [34] using bovine serum albumin as standard.

Thiobarbituric acid-reactive substances (TBARS) were measured in the supernatant of liver tissue homogenates as described by Tappel and Zalkin, 1959 [35]. The color intensity of the TBARS reactants as malondialdehyde was measured at 532 nm and a molar extinction coefficient of 156,000 M^−1 ^cm^−1^ was used for calculation of the TBARS concentration.

#### 2.3.1. Total Cholesterol Level

Cholesterol was determined enzymatically with cholesterol esterase and cholesterol oxidase. Cholesterol esters are cleaved by the action of cholesterol esterase to yield free cholesterol and free fatty acids. The color intensity is directly proportional to the concentration of cholesterol and was determined spectrophotometrically. The full procedure of cholesterol determination was followed according to the instructions of the kit provided by Biosystems, Barcelona, Spain.

#### 2.3.2. Lipid Determinations

Briefly, HDL was measured in the plasma after precipitation of apolipoprotein B-containing particles with dextran sulfate-MgCl_2_. For LDL, a specific detergent solubilizes the cholesterol from HDL, very low-density lipoproteins and chylomicrons. The cholesterol esters are broken down by cholesterol esterase and cholesterol oxidase in a non-color-forming reaction. The second detergent (MES buffer, >30 mmol/l, N,N-bis(4-sulfobutyl)-m-toluidine 1 mmol/l, detergent, pH 6.3) solubilizes cholesterol from LDL in the sample, and LDL-cholesterol is then measured spectrophotometrically at 546 nm. The full procedure of HDL- and LDL-cholesterol determination was followed according to the instructions of kits provided by Biosystems, Barcelona, Spain.

#### 2.3.3. Homocysteine Determination

Most homocysteine in plasma is bound to proteins by disulfide bonds with thiol-containing residues; oxidized homocysteine and homocysteine-cysteine mixed disulfide are also present. Total homocysteine was measured after reduction of disulfide bonds and detection of released homocysteine by use of dithiothreitol. Homocysteine in test samples is converted to S-adenosyl-L-homocysteine (SAH) by use of S-adenosyl-L-homocysteine hydroxylase and excess adenosine. The subsequent solid-phase enzyme immunoassay is based on competition between SAH in the sample and immobilized SAH bound to the walls of the microtiter plate for binding sites on a monoclonal anti-SAH antibody. After removal of anti-SAH antibody not bound to the plate, a secondary rabbit anti-mouse antibody labeled with horseradish peroxidase is added. Peroxidase activity is measured spectrophotometrically after addition of the substrate, and the absorbance is inversely related to the concentration of total homocysteine in the sample [36]. The full procedure for homocysteine determination was followed according to the instructions for the homocysteine enzyme immunoassay kit (Axis-Shield, Germany).

#### 2.3.4. Superoxide Dismutase Assay

SOD was measured according to the method of Beers and Sizer, 1952 [37]. SOD estimation is based on the generation of superoxide radicals produced by xanthine and xanthine oxidase, which react with nitroblue tetrazolium to form formazin dye. SOD activity is then measured at 560 nm by the degree of inhibition of this reaction and expressed as mmol/min/mL of plasma.

#### 2.3.5. Glutathione Peroxidase

Glutathione peroxidase enzyme activity (GPx; EC. 1.11.1.9) was assayed according to the method of Chiu et al., 1976 [38]. The reaction mixture (1 mL) containing 0.05 mL of the enzyme source, 0.05 M Tris-HCl buffer (pH 7.6), 1.5 mM GSH, and cumenehydroperoxide was incubated for 5 min at 37°C. In another tube, the control sample was prepared without cumenehydroperoxides and incubated for 5 min at 37°C. To both control and test samples, 1.0 mL of TCA (15%) was added, while 0.1 mL cumenehydroperoxide was added to the control only. Both tubes were incubated for 10 min at 37°C and centrifuged at 3000 rpm for 20 min. Tris-HCL buffer (pH 8.9) and 1.5 mM DTNB were added to 1 mL supernatant for both sample and control. The optical density of the yellow color obtained was measured at 412 nm within 5 min. Result was expressed as U/gm tissue.

#### 2.3.6. Catalase Activity

Catalase activity was determined by the method by Beers and Sizer, 1952 [37], which is based on decreasing absorbance of H_2_O_2_ solution decomposed by the enzyme, with a kit obtained from Randox Laboratory, UK. The quantity of H_2_O_2_ decomposed over a specified time is calculated from the molar absorbance coefficient. Absorbance is measured at 240 nm and catalase activity is expressed as IU/gm tissue.

#### 2.3.7. Creatine Kinase MB

A specific antibody inhibits the M subunits of CK MM and CK MB and thus allows determination of the B subunit of CK MB (assuming absence of CK BB and CK 1); Ck B catalytic concentration which responds to half of CK MB concentration is determined by rate of NADPH formation, measured at 340 nm by means of hexokinase and glucose 6 phosphate dehydrogenase coupled reactions. The full procedure of creatine kinase MB determination was followed according to the instructions with the kit (spectrum diagnostics, Hannover, Germany).

#### 2.3.8. Lactate Dehydrogenase Determination

Lactate dehydrogenase LDH catalyzes the reduction of pyruvate by NADH to form lactate and NAD+. The catalytic concentration is determined from the rate of decrease of NADH measured at 340 nm. The full procedure of lactate dehydrogenase determination was followed according to the instructions of the kit (Biosystems, Barcelona, Spain).

#### 2.3.9. Paraoxonase Activity

Paraoxonase activity was measured according to method of Eckerson et al., 1983 [39], with 1 mM paraoxon in total volume of 800 *μ*L. Enzyme activity was measured in 50 mM Tris-HCL buffer. Non-enzymatic assay mixture contained 600 *μ*L buffer and 200 *μ*L paraoxon as substrate, while enzymatic assay mixture contained enzyme to start the reaction. The amount of formed p-nitrophenol was measured spectrophotometrically at 412 nm. The enzymatic activity was calculated using 12,800 M^−1^ Cm^−1^ as molar extinction coefficient. Blank contained substrate without enzyme, one unit of paraoxonase activity defined as 1 nmole of 4-nitrophenol formed per min under the above conditions [23].

#### 2.3.10. Western Immunoblotting

From pooled sample of each treatment, 20 *μ*g of S-9 liver supernatant proteins was prepared and subjected to SDS-polyacrylamide gel electrophoresis. Proteins were transblotted to Hybond-C nitrocellulose membrane (Amersham, UK). GST *π* isozyme and glutathione peroxidase were visualized after binding with their specific monoclonal antibodies. Chemiluminescence signals of both were detected according to the manufacturer's instructions (Abcam, UK) [40].

### 2.4. Statistical Analysis

Means and standard errors were calculated. Data were analyzed by one-way analysis of variance (ANOVA) compared with Student's *t*-test. The level of significance for all experiments was set at *P* < 0.05.

## 3. Results

The present study showed that the level of cholesterol increased by 13, 20, and 16% after treatment of male rats for two weeks as repeated doses with diethyl-, dimethyl-, and diphenylnitrosamine, respectively, while other compounds have no significant effects compared to control group (Table 1). On the other hand, the level of HDL decreased by 24, 27, 32, 31, and 33% and LDL level increased by 18, 44, 20, 29, and 27% and paraoxonase activity increased by 84, 40, 81, 121, and 48% in plasma of rats after treatment with ethylbutyl-, butylpropyl-, diethyl-, dimethyl-, and diphenylnitrosamine, respectively, compared to control group (Table 1).

**Figure 1:**
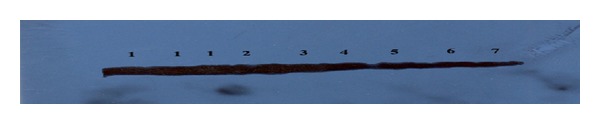
Western immunoblot analysis showed the protein expression of glutathione peroxidase. Lanes 1, 2, 3, 4, 5, 6, and 7 represent the pooled proteins of matched control, diphenylnitrosamine, dimethylnitrosamine, diethylnitrosamine, butylpropylnitrosamine, ethylbutylnitrosamine, and dibutylnitrosamine-treated groups, respectively.

Biomarkers of cardiovascular diseases, creatine kinase MB activity, increased by 86, 71, 91, 100, and 103% after rat treatment with dibutyl-, ethylbutyl-, butylpropyl-, diethyl-, and dimethylnitrosamine whereas diphenylnitrosamine has no significant effect compared to control group (Table 1). In addition, the activity of lactate dehydrogenase increased by 44 and 50% after treatment with dibutyl- and diethylnitrosamine while the other nitrosamines have no significant effects compared to control group (Table 1). Homocysteine level is increased in rat plasma by 25% after treatment with dibutylnitrosamine and decreased by 19 and 25% after treatment with ethylbutyl- and dimethylnitrosamine, respectively, while the other nitrosamines have no significant effect compared to control group (Table 1). Treatment of rats with dibutyl-, ethylbutyl-, butylpropyl-, diethyl-, and dimethylnitrosamine increased both reduced GSH level and GR activity by 25, 20, 23, 22, and 39% and 100, 44, 34, 55, and 54%, respectively (Table 2). Glutathione peroxidase activity decreased by 69, 71, 70, 57, 43, and 15% after treatment of rats with dibutyl-, ethylbutyl-, butylpropyl-, diethyl-, dimethyl-, and diphenylnitrosamine, respectively (Table 2), whereas treatment of rats with dibutyl-, ethylbutyl-, and dimethylnitrosamine increased catalase activity by 131, 169, and 342%, respectively (Table 2). In addition, the protein expression of glutathione peroxidase was also reduced after treatment of rats with diethylnitrosamine, butylpropylnitrosamine, ethylbutylnitrosamine, and dibutylnitrosamine for the same period (Figure 1).

**Figure 2:**
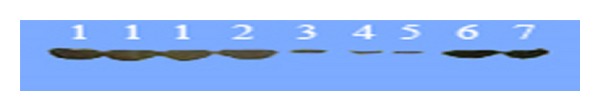
Western immunoblot analysis showed the protein expression of glutathione S-transferase *π* isozyme. Lanes 1, 2, 3, 4, 5, 6, and 7 represent the pooled proteins of matched control, diphenylnitrosamine, dimethylnitrosamine, diethylnitrosamine, butylpropylnitrosamine, ethylbutylnitrosamine, and dibutylnitrosamine-treated groups, respectively.

The activity of total GST increased by 54, 60, and 51% after treatment of rats with dibutyl-, ethylbutyl-, and diethylnitrosamine, respectively, and decreased by 36% after treatment with diphenylnitrosamine (Table 2). The western immunoblotting of glutathione S-transferase pi isozymes was markedly inhibited after treatment of rats with diethylnitrosamine, butylpropylnitrosamine, ethylbutylnitrosamine, and dibutylnitrosamine for two weeks (Figure 2). The levels of malondialdehyde, indicator of free radical, increased by 98, 52, 45, and 131% after treatment with dibutyl-, ethylbutyl-, butylpropyl-, and dimethylnitrosamine, respectively (Table 2). On the other hand, SOD activity decreased by 48, 52, and 52% by dibutyl-, butylpropyl-, and diphenylnitrosamine, respectively, compared to control (Table 2).

## 4. Discussion

Hypercholesterolemia (HC), especially high levels of low-density lipoprotein-cholesterol (LDL), is an important risk factor accounting for severe atherosclerotic diseases [41]. When LDL enters into the endothelium of vessel walls by a concentration-dependent mechanism, reactive oxygen species (ROS) exert oxidative attack on lipid and protein components of LDL generating oxidized LDL (LDLox) [42, 43]. Additionally, LDLox can initiate and enhance the inflammatory process, which plays a vital role in the development of atherosclerotic changes [44]. In accordance with these findings, the present study showed that LDL levels have been increased after treatment of rats with all tested N-nitrosamines except NDBA did not change such level (Table 1). It is well known that conversion of HDL into LDL is mediated via different mechanisms, and free radicals are involved in such conversion [42, 43]. In agreement with these findings, levels of both generated free radicals and LDL were markedly increased, whereas HDL levels decreased after treatment of rats with different types of N-nitrosamines (Table 1). It is thought that paraoxonase enzyme protects HDL against oxidation which mediated via reactive oxygen species [19, 45]. However, in the present study, paraoxonase could not protect HDL from oxidation since its activity was potentially induced and HDL levels decreased after treatment of rats with various types of N-nitrosamines (Table 1). This discrepancy might be due to the presence of more than one type of paraoxonase isozyme [19]. In the present study, the total activities of all types of paraoxonase were measured but not the specific activity of paraoxonase-1 which is associated with HDL.

It is well known that high levels of free radicals lead to oxidative stress, which is involved in the initiation and progression of different types of cardiovascular diseases [19, 46, 47]. Supporting our finding, CVD biomarker, CK-MB activity, has been markedly increased in the plasma of N-nitrosamines-treated rats (Table 1). From the present study, LDH enzyme is not specific biomarker for CVD since its activity did not change in plasma of treated rats except NDBA and NDEA increased such activity (Table 1). Therefore, this study could provide a new possible reason for the incidence of CVDs among people who are exposed to these compounds from different environmental sources as tobacco smoke and cured foods.

It is well known that increasing level of homocysteine is a risk factor for cardiovascular disease and plays a significant role in the incidence of CVD [48] via different mechanisms including endothelial dysfunction, superoxide and hydrogen peroxide generation, and promoted proliferation of the aortic smooth muscle cells. However, in the present study, the level of homocysteine did not change after treatment of rats with most of N-nitrosamines except NEBA and NDMA decreased such level (Table 1). Therefore, it is suggested that N-nitrosamines could induce the incidence of CVDs via a mechanism other than increasing of homocysteine levels which might be due to generation of high levels of ROS which are linked to the damage of endothelial lining of arterial vessels [46, 47, 49]. ROS induce specific posttranslational modifications that alter the function of important cellular proteins and signaling pathways in the heart [50–54].

It is well known that antioxidant enzymes such as superoxide dismutase, glutathione peroxidase, glutathione reductase, and catalase play a significant role in the removal of free radicals such as active superoxide and hydroxy radicals from different tissues. Therefore, in the present study, increasing free radical levels after treatment of rats with different N-nitrosamines is mainly due to inhibition of antioxidant enzymes such as superoxide dismutase and glutathione peroxidase (Table 2). In addition, protein expression of glutathione peroxidase decreased after treatment of rats with most of N-nitrosamines (Table 2 and Figure 1). Activity of antioxidant enzymes such as catalase, glutathione peroxidase-1 (GPX-1), and SOD has been quantified in plasma as measures of antioxidant capabilities. In a prospective study of patients with suspected coronary artery disease, erythrocyte GPX-1 and SOD activity were inversely associated with incidence of cardiovascular events after adjusting risk factors of cardiovascular diseases [55, 56].

The glutathione (GSH) and GSH S-transferases system are major players in protection against a broad variety of toxic agents involved in the pathogenesis of several chronic degenerative diseases [57–60]. The present study provides further evidence to these findings since the protein expression of GST *π* was potentially reduced after treatment of rats with dimethylnitrosamine, diethylnitrosamine, butylpropylnitrosamine, ethylbutylnitrosamine, and dibutylnitrosamine (Figure 2). It seems from this study that the chemical structure of these compounds plays a significant role in the modification of GST expression since the substitution of the diphenyl by diethyl, ethylbutyl, and/or dibutyl on the nitroso group changed the activity and the expression of GST. The inhibition of GST activity could potentiate the toxicity of environmental carcinogens and might increase the probability of DNA binding with the ultimate metabolites of well-known carcinogens. Supporting our finding, it has been found that DNA adducts of GSTM1 gene in smooth muscle cells (SMC) of human abdominal and thoracic aortic samples were higher in subjects with frequent atherosclerotic changes as compared to subjects with rare lesions [61, 62]. In addition, the level of GSH was increased after treatment of rats with all tested N-nitrosamines (Table 2). It is taught that increment in GSH might be due to induction of GSH synthase and gamma-glutamyltransferase [63–65]. However, in the present study, the increased level of GSH was found to be dependent on the activity of GR since the activity of GR was also potentially induced after treatment of rats with N-nitrosamines (Table 2). These increments in both GSH levels and GR activity might be a defense mechanism of the treated animals to reduce the toxicity of N-nitrosamines [66].

It is concluded that N-nitrosamines could induce CVDs via increasing free radical, LDL levels and inhibition of antioxidant enzyme activities as well as decreasing HDL levels. In addition, the protein expressions of both GST *π* and glutathione peroxidase were decreased after exposure to these compounds. CVDs biomarker, CK-MB activity, was also potentially induced, and these changes might explain the implication of these compounds in the initiation and progression of CVD. Also, this study might provide an explanation for the association of tobacco smokers with CVDs since tobacco is a major risk factor for cardiovascular disease due to the presence of these compounds in large quantities [67].

## Figures and Tables

**Table 1:** Changes in levels of total cholesterol, LDL, HDL, homocysteine and activities of lactate dehydrogenase and creatine kinase MB after treatment of male rats with a daily dose of dibuty-, ethylbutyl-, butylpropyl-, diethyl-, dimethyl- and diphenylnitrosamine for two weeks.

	Control	NDBA	NEBA	NBPA	NDEA	NDMA	NDPA
Total cholesterol [mg/dL]	97.0 ± 3.59	97.0 ± 4.202 Ns	99.0 ± 3.929 Ns	94.5 ± 3.687 Ns	110.0 ± 3.325 (+13%, *P* < 0.05)∗	116.0 ± 3.733 (+20%, *P* < 0.05)∗	112.0 ± 5.529 (+16%, *P* < 0.05)∗

LDL-Cholesterol [mg/dL]	181.0 ± 8.56	207.0 ± 6.71 Ns	214.0 ± 12.47 (+18%, *P* < 0.05)∗	260.0 ± 11.88 (+44%, *P* < 0.05)∗	217.0 ± 8.91 (+20%, *P* < 0.05)∗	233.0 ± 12.14 (+29%, *P* < 0.05)∗	229.0 ± 15.32 (+27%, *P* < 0.05)∗

HDL-Cholesterol [mg/dL]	59.0 ± 2.9	48.5 ± 2.59 (−18%, *P* < 0.05)∗	45.0 ± 2.01 (−24%, *P* < 0.05)∗	42.9 ± 1.95 (−27%, *P* < 0.05)∗	40.0 ± 1.69 (−32%, *P* < 0.05)	40.5 ± 1.89 (−31%, *P* < 0.05)∗	39.0 ± 2.45 (−33%, *P* < 0.05)∗

Lactate dehydrogenase [Units/L]	428.0 ± 34.5	617.0 ± 42.12 (+44%, *P* < 0.05)∗	409.7 ± 33.37 Ns	369.5 ± 28.43 NS	640.0 ± 95.64 (+50%, *P* < 0.05)	530.0 ± 54.77 Ns	508.0 ± 23.47 Ns

Creatine kinase MB [Units/L]	411.0 ± 47.1	764.0 ± 51.22 (+86%, *P* < 0.05)∗	703.0 ± 48.08 (+71%, *P* < 0.05)∗	787.0 ± 52.13 (+91%, *P* < 0.05)∗	822.0 ± 60.60 (+100%, *P* < 0.05)∗	833.0 ± 53.45 (+103%, *P* < 0.05)	543.5 ± 54.59 NS

Homocysteine *μ*mole homocysteine/L	11.78 ± 0.692	14.76 ± 1.108 (+25%, *P* < 0.05)∗	9.5 ± 0.358 (−19%, *P* < 0.05)∗	9.98 ± 0.395 Ns	11.84 ± 0.963 Ns	8.84 ± 0.227 (−25%, *P* < 0.05)∗	10.6 ± 0.496 Ns

Values are expressed as Mean ± SEM.

NS: values are not significant statistically compared to control group where *P* > 0.05.

∗Values are significant compared to control group where *P* < 0.05.

**Table 2:** Changes in the activities of superoxide dismutase, GSH-R, GST, paraoxonase, catalase, glutathione peroxidase and levels of both GSH and free radicals after treatment of male rats with a daily doses of dibuty-, ethylbutyl-, butylpropyl-, diethyl-, dimethyl- and diphenylnitrosamine for two weeks.

	Control	NDBA	NEBA	NBPA	NDEA	NDMA	NDPA
GSH content *μ*mole/gm liver	1.8165 ± 0.39	2.268 ± 0.724 (+25%, *P* < 0.05)∗	2.184 ± 0.11 (+20%, *P* < 0.05)∗	2.227 ± 0.836 (+23%, *P* < 0.05)∗	2.21 ± 0.10 (+22%, *P* < 0.05)∗	2.516 ± 0.682 (+39%, *P* < 0.05)∗	2.091 ± 0.49 (+15%, *P* < 0.05)∗

Free Radicals (TBARS) *μ*mole/gm liver	5.481 ± 0.35	10.856 ± 1.24 (+98%, *P* < 0.05)∗	8.35 ± 0.531 (+52%, *P* < 0.05)∗	7.952 ± 0.421 (+45%, *P* < 0.05)∗	7.552 ± 0.865 Ns	12.68 ± 1.427 (+131%, *P* < 0.05)∗	7.618 ± 0.42 Ns

GSH-R activity nmolNADPH/mgprotein/min	135 ± 5.12	269 ± 8.75 (+100%, *P* < 0.05)∗	194 ± 8.4 (+44%, *P* < 0.05)∗	181 ± 10.92 (+34%, *P* < 0.05)∗	208 ± 7.22 (+55%, *P* < 0.05)∗	208 ± 13.84 (+54%, *P* < 0.05)∗	139.39 ± 7.22 Ns

GST activity mmole/mgprotein/min	0.335 ± 0.008	0.515 ± 0.023 (+54%, *P* < 0.05)∗	0.536 ± 0.016 (+60%, *P* < 0.05)∗	0.364 ± 0.199 Ns	0.506 ± 0.027 (+51%, *P* < 0.05)∗	0.361 ± 0.013 Ns	0.215 ± 0.006 (−36%, *P* < 0.05)∗

Superoxide dismutase Units/g hemoglobin	1.603 ± 0.231	0.837 ± 0.186 (−48%, *P* < 0.05)∗	1.460 ± 0.209 Ns	0.769 ± 0.096 (−52%, *P* < 0.05)∗	1.985 ± 0.065 Ns	1.20 ± 0.194 Ns	0.772 ± 0.161 (−52%, *P* < 0.05)∗

Paraoxonase activity *μ*mole Paraoxonhydrolyzed/mg protein/min	8.249 ± 0.600	9.527 ± 1.11 Ns	15.173 ± 1.469 (+84%, *P* < 0.05)∗	11.577 ± 0.823 (+40%, *P* < 0.05)∗	14.90 ± 0.773 (+81%, *P* < 0.05)∗	18.234 ± 0.584 (+121%, *P* < 0.05)∗	12.22 ± 0.797 (+48%, *P* < 0.05)∗

Catalase (U/gm tissue)	3.05 ± 0.32	7.01 ± 0.56 (+131%, *P* < 0.05)	8.14 ± 0.43 (+169%, *P* < 0.05)	2.39 ± 0.18 NS	2.02 ± 0.22 NS	13.4 ± 0.61 (339%, *P* < 0.05)	2.86 ± 0.36 NS

Glutathione peroxidase IU/gm Wet tissue	69.29 ± 1.94	21.7 ± 1.11 (−69%, *P* < 0.05)∗	20.41 ± 0.99 (−71%, *P* < 0.05)∗	21.04 ± 0.6 (−70%, *P* < 0.05)∗	29.8 ± 0.75 (−57%, *P* < 0.05)∗	39.83 ± 2.26 (−43%, *P* < 0.05)∗	59 ± 2.93 (−15%, *P* < 0.05)∗

Values are expressed as Mean ± SEM.

NS: values are not significant statistically compared to the control group where *P* > 0.05.

∗Values are significantly differentcompared to control group where *P* < 0.05.
